# Supplementary figures and images for: Search for Transcriptional and Metabolic Markers of Grape Pre-Ripening and Ripening and Insights into Specific Aroma Development in Three Portuguese Cultivars
Source: PLoS One. 2013 Apr 2;8(4):e60422. doi: 10.1371/journal.pone.0060422 (PMC3614522; doi:10.1371/journal.pone.0060422)

## Slide 1
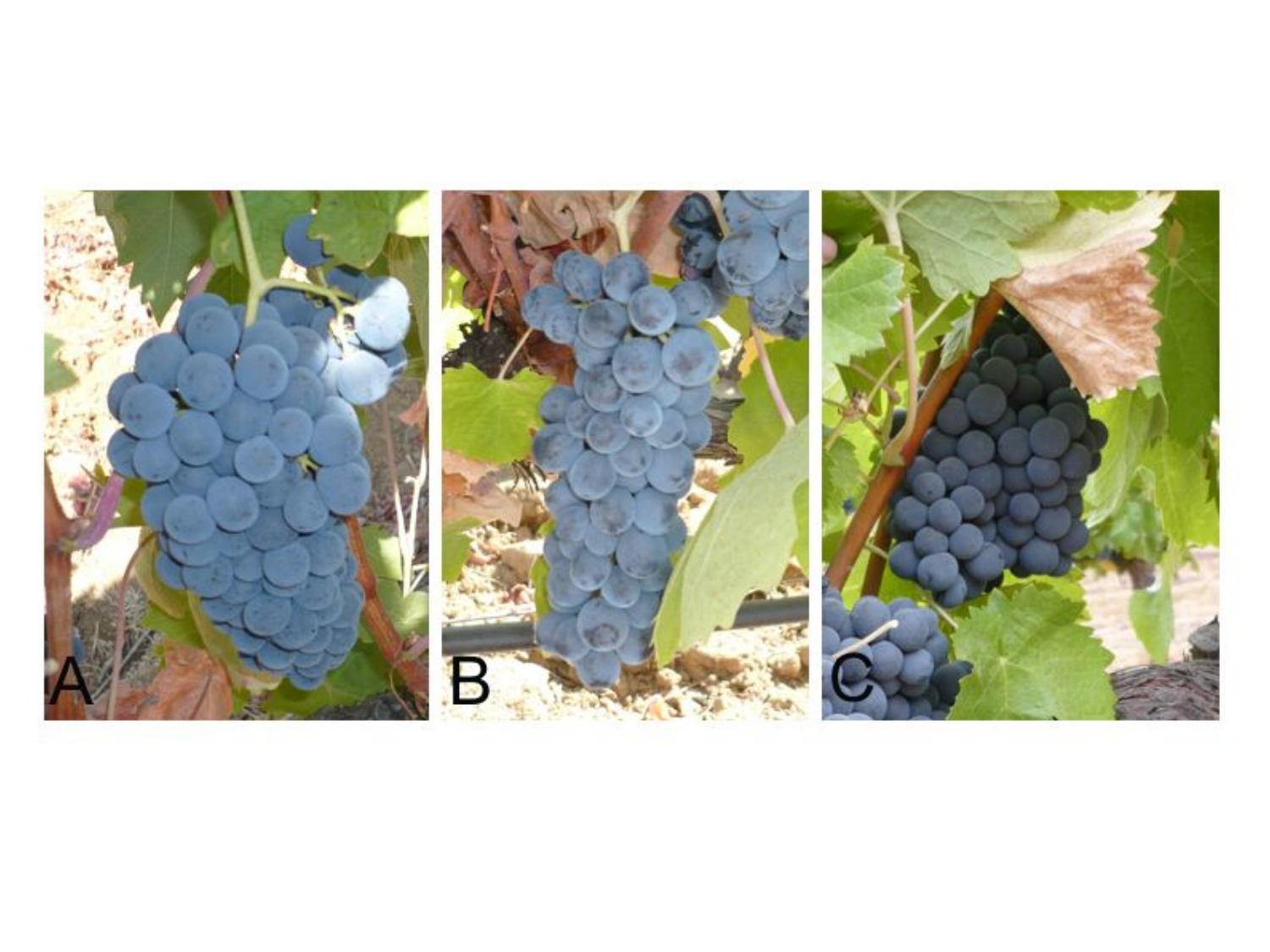

Supplement: Figure S1 — Samples of berries at EL 38 stage of development. A Aragonês B Touriga Nacional C Trincadeira. (PPTX) [file pone.0060422.s001.pptx]

## Slide 1
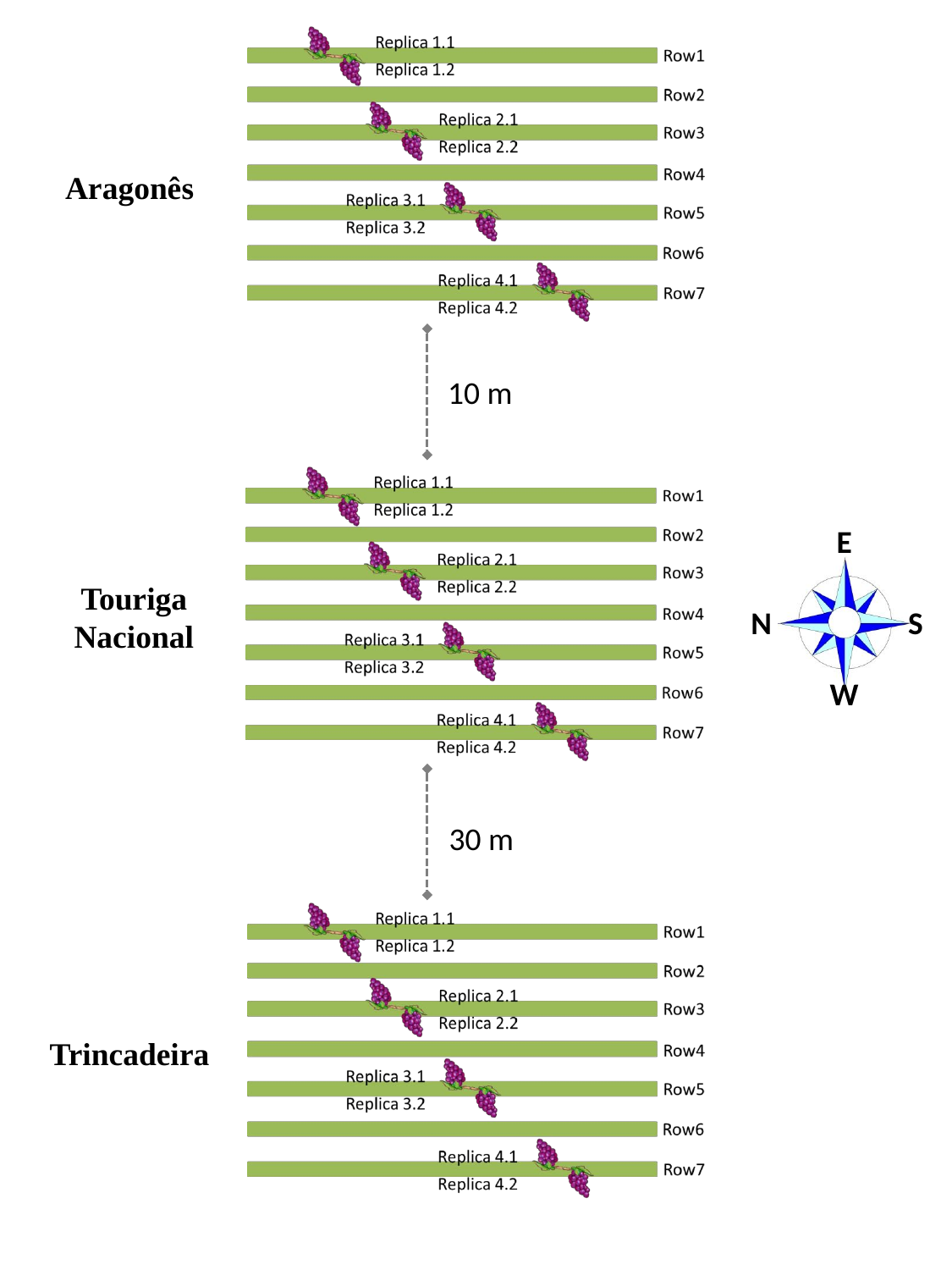

Aragonês
10 m
E
W
N S
Touriga
Nacional
30 m
Trincadeira

Supplement: Figure S2 — Sketch of the sampling layout. (PPTX) [file pone.0060422.s002.pptx]
