# Supplementary material for: Search for Transcriptional and Metabolic Markers of Grape Pre-Ripening and Ripening and Insights into Specific Aroma Development in Three Portuguese Cultivars
Source: PLoS One. 2013 Apr 2;8(4):e60422. doi: 10.1371/journal.pone.0060422 (PMC3614522; doi:10.1371/journal.pone.0060422)
Supplement: Table S1 — List of primers used in real time reverse transcription-polymerase chain reaction. (DOCX) [file pone.0060422.s003.docx]

**Table S1**

| **Target sequences** | **Probe ID** |  | **5’ to 3’** | **Tm** | **% GC** | **Length** |
| --- | --- | --- | --- | --- | --- | --- |
| **Actin** | VVTU17999_s_at | Fwd | **GGTCAACCATGTTCCCTGGTATT** | 59.8 | 48 | 23 |
|  |  | Rev | **GGAGCAAGAGCAGTGATTTCCTT** | 59.9 | 48 | 23 |
| **Succinic semialdehyde dehydrogenase (SSADH1)** | VVTU35625_s_at | Fwd | **TAGAGGCTTGTGCTTGGATTTTT** | 58.1 | 39 | 23 |
|  |  | Rev | **CGCAACCCCGCAACA** | 58.1 | 67 | 15 |
| **L-galactono-1,4-lactone dehydrogenase (LGDH)** | VVTU8069_at | Fwd | **TGGAGAAAGTCAGAGGGATACAGA** | 58 | 46 | 24 |
|  |  | Rev | **CACCACAATCAAATCCCAAGATT** | 58.7 | 39 | 23 |
| **Flavonol synthase (FLS)** | VVTU16103_at | Fwd | **CTTGGAGCCACCGTCTGATC** | 59.2 | 60 | 20 |
|  |  | Rev | **TTCTTGGTCTTGTACCTAGCAGGAT** | 58.5 | 44 | 25 |
| **Limonoid UDP-glucosyltransferase** | VVTU17111_s_at | Fwd | **AGTCACCGACGCCAAGTACCT** | 59.6 | 57 | 21 |
|  |  | Rev | **CACATTCTCACCCCAACTTTGAA** | 59.8 | 43 | 23 |
| **Caffeic acid**  **O-methyltransferase (COMT)** | VVTU36927_x_at | Fwd | **ATGGAGTGATACGAGAAGATAGCGAT** | 59.5 | 42 | 26 |
|  |  | Rev | **TCAGTAAATCGAACACCAACCTTGT** | 59.8 | 40 | 25 |
| **Cytochrome P450 monooxygenase (CYP89H3)** | VVTU21329_at | Fwd | **TCAAGAACCTCTCAGGCTTGAACT** | 59.5 | 46 | 24 |
|  |  | Rev | **CACTTTGTGAGCCCTCACTCAGT** | 59.4 | 52 | 23 |
| **Palmitoyl-monogalacto_ syldiacylglycerol delta-7 desaturase, chloroplast** | VVTU3709_at | Fwd | **CTTTTGAATACTCCGCTCGTCAT** | 58.5 | 43 | 23 |
|  |  | Rev | **TGAAGGAACCTAACCACGTACCA** | 59.4 | 48 | 23 |
| **Tocopherol cyclase (TC)** | VVTU22626_s_at | Fwd | **CACCAGAGCTTGTTCGCCTT** | 59.1 | 55 | 20 |
|  |  | Rev | **TAGAAGGCTTGGCGAGACCA** | 59.5 | 55 | 20 |
| **Terpene synthase (VvTPS10)** | GSVIVT01036344001 | Fwd | **TAGAATACCCTCTTGCAGCACAAGTA** | 59.4 | 42 | 26 |
|  |  | Rev | **ATGTGACGCTTCATCCTGGTAGATA** | 59.9 | 44 | 25 |
| **Terpene synthase (VvTPS34)** | GSVIVT01000401001 | Fwd | **TGATGGCAGCGTAAAGGAGC** | 59.9 | 55 | 20 |
|  |  | Rev | **AAGTGTCAATGGCTCTGCACTCT** | 59.1 | 48 | 23 |
